# A Hospital in the Black Forest

**Published:** 1890-08-30

**Authors:** 


### A Hospital in the Black Forest.

(By a Foreign Correspondent.)

Freiburg, in Baden, is a town of some 40,000 inhabitants; famous in the past from its position in the marches between France and Germany; celebrated for its surroundings in the Black Forest, which have made it, to some extent, a residential town; and interesting to the professional man from its medical school and laboratories. The university is returned in the latest calender of the German universities as having 98 teachers and 1,233 students, of whom 452 are medical.

It will be found by the inquirer that the web of medical and scientific education and practice is much the same among civilized nations, however the shades and colouring may vary; and the readers of The Hospital will desire rather to have what is new and different from their wont presented to them, than to be told that the hospital is a large, oblong building of stone, and that the sick are laid in bed much as they are laid in bed at home. There are hospital sisters in Freiburg as there at home, dressed in plain black gowns, with white headgear carrying broad wings, and with some of the symbols of the Romish persuasion about their dress. A few of them served in the war of 1870-71. They tell of a railway accident which sent 120 wounded to their care in one night; but that was nothing to the days of the war, when hundreds of dead and dying were hurried to them in a few hours time.

And what do you think of war?

Oh! I am for peace! If I were a queen I should be for peace! But, after all, I would sooner be a sister of mercy than any queen.

Yes; you are devoted to your calling; and such sisters we have in England, too.

The various component parts of the university are located in separate buildings. There is a Chemical Institute, a Hygienic and Pathological Institute, and a good many more, either complete or in process of erection. One handsome building is devoted to the surgical clinic with the out-patient room, in which patients are received at all hours of the day and night, on the ground floor. Cleanliness, lighting, and arrangements of the room are alike admirable. Rings in the roof are used for rope and pulley in cases of spinal curvature. The principal antiseptics employed are corrosive sublimate and carbolic acid; but wools, such as salicylic, are also at hand. All bandages and dressings are rendered aseptic with sublimate in the house. All sponges are kept in jars, labelled with the days of the week, containing 5 per cent. carbolic acid. A sponge used on Monday is replaced, after cleansing, in the jar marked Monday, and will not be used again for another eight days.

The principal operation theatre is close by; all operations, however, upon infectious cases, such as erysipelas and tracheotomies in diphtheria, are done in a separate building. The room is semi-circular, resembling the nave of a church, very handsome, with lofty windows in the bow and a dome of glass in the roof. The operator will have in front the windows, to right and left his senior students, while junior students are located in a gallery. The operation-table runs on wheels, so that it may be wheeled round; instruments are wheeled in on other tables. Before use, all instruments are boiled in 2 per cent. carbolic acid for half an-hour in simple copper pans, and are then exposed to steam in a boiler. A shaft in a convenient situation leads down to the cellars, and into this all dressings and soiled materials are at once thrown to be destroyed. Under this roof there are no patients, if we except a few children, delighted with the promise of some Christmas trees. Their complaints are principally of a tubercular nature.

Across the roadway stands a much larger building containing the bulk of the medical and surgical patients. The present hospital was originally a barracks, and to an eye accustomed to the more modern and better regulated hospitals of London would appear rather dingy and confined. The kitchens are located here, so that food has to be conveyed across the road to the surgical clinic. Such an underground passage, as visitors to Brompton Consumption Hospital are familiar with, is talked of, to be constructed under the roadway. Included within the precincts of this building are a large lecture theatre, where operations upon all infectious cases are done, so that there may be no risk of infection in the ordinary operating theatre, the various domestic offices just referred to, and the medical and surgical wards. Ventilation is partially effected in some of these wards by means of openings in the roof, which communicate with chambers having windows opening outwards. One patient was suffering from a large goître without hereditary history, a disease, however, not so common in Freiburg as in the adjacent Switzerland; another had fractured one of the small bones of the toes. There was a case of rectal disease treated by a new operative procedure, which calls for comment. In England it is not usual to operate in cases of rectal disease extending above the peritoneal reflection. Here in these cases surgeons do not shrink from operating even to an inch higher by a new operation which involves total removal of the coccyx, and removal of the left wing of the sacrum as high as the third foramen. The sacral canal is in no case opened. Ample space is thus obtained for reaching the pelvic organs, and it has been proposed to extend this procedure to gynecological cases. In cases convalescent after operation it is surprising how insignificant the scar in the sacral furrow is. Another novelty in another direction is a new filling material for operative holes made in bone. Bone itself is used. Fresh bones are steeped in dilute acid so that they become fully decalcified, are then dried, and then handed over to the turner, who converts them into shavings. The shavings after treatment with solution of corrosive sublimate, alcohol, and powdering with iodoform are ready for use. A similar appliance is decalcified long bones of young horses and birds, which are used for drainage purposes. Both these novel inventions deserve a trial in England. In the latter case one has what is tantamount to a pipe of gristle, which is passed into and drains the wounds, is unirritating and capable of absorption.

The next ward was a medical ward containing some ten beds, divided off from its neighbour however, only by a partition completed by curtain hangings, leaving one room to pass. These curtains had a pleasing effect, much as the red, green, and blue tiles on the roof of a church outside. Possibly a lesson is conveyed to some English hospitals with their long, dreary, cold wards with white-washed walls, which must exert a very depressing influence upon the patients. There were no less than four cases of typhoid fever in the first few beds, and it appeared that Freiburg, equally with the adjacent town of Basel, has more of this malady than the average. The treatment which has been found most successful is bathing in cold water. Whenever the temperature reaches 103°, the patient is bathed, it may be as many as eight times a day. He may remain in the bath as long as ten minutes, the temperature of the water being 70°. Each bath, containing a pillow for the patient's head, is wheeled alongside the bed; the patient may help himself in, or assistance may have to be rendered. Drugs, such as hydrochloric acid, are not relied upon, though calomel has been given during the first week as an abortive, in doses of 10 grains a day. Antipyrine is not used as an antipyretic.

Among other cases were one of bronchiectasis being treated with myrthol in capsules, 1½ grains four times a-day. Operative measures for making a free opening into the chest in cases of bronchiectasis and phthisical vomicæ have not been practised in Freiburg. A case of spastic paraplegia in a woman was doing well with galvanism, saltwater baths, and iron. The appearance of the galvanic machine, a large cabinet running on wheels with every elegance of arrangement for faradizing and galvanizing, would shame most of those we have at home. Electrical treatment is only resorted to so long as the nerve reacts. There was a case of multiple peripheral neuritis in a male, with remarkable hyperæsthesia. Diuretics are not relied upon in pleurisy. A very neat inhalation apparatus of Schreiber deserves notice. The water containing the medicament is volatilized in a small boiler heated by a spirit lamp. The dispensary is kept in beautiful order, the bottles beautifully and artistically labelled.

"You rely upon your drugs, I see?"

"No," said my informant. "We don't prescribe as much as you do in England."

One is somewhat surprised on thinking of the many new drugs yearly announced from Germany.

The Eye Institute is a convenient building, again separate, having on the ground floor a large room used for the reception of patients, as well as for teaching purposes; from this opens the "dark-room," which serves also as a storehouse for microscopic preparations and sections and microtomes; yet further being the house-surgeon's apartments. Among unfamiliar drugs upon the table were hyoscine, a more powerful nydriatic than atropine; and two antiseptics, at least principally such—creoline, allied to carbolic acid, and höllenstein. Among instruments were the old familiar perimeter, and a new instrument like a binocular microscope, placed horizontally, for looking at the patient's eyes, which are coincidently lighted from the side with the help of condensing lenses. Upstairs are the wards, each containing some six or seven beds, except the cataract ward for patients after operation, which contained but three. All these wards are dark; light and air are admitted through one pane space, from which the pane has been removed. The small ward is kept quite dark. Clinically there was nothing of interest. It will, however, reassure readers given over to indulgence in tobacco to be informed that nothing is known in especial of a tobacco amblyopia, although the Germans are justly reported to be great smokers of cigars.

In this part of the Continent breakfast consists of coffee with bread, which the patients have at nine a.m., with bouillon in addition. They dine at noon, off soup, meat, and vegetables, or fruit; in the afternoon they have coffee, and at half-past six partake of the evening meal, which may include some cold meat.

While it will be thus apparent that the ordinary hospital arrangements and treatment of the sick are conducted pretty generally as in England, it is to be noted that the student has far more supervision, has every appliance for learning and research, and is not left so much as in London to sharpen his own axe. Without adducing more instances, or entering the Hospital for women, also in a separate building, or the Pathological and Hygienic Institution, with its hundreds of tubes of Bacteria cultivations, it must be evident that lecture-room, teacher, and student are brought very near the patient; that every mechanical and artistic help for the student are present; and that the accepted axiom of the land, that a Hospital is for the training of the doctor, as well as for the curing of the sick man's malady, is never left out of sight.

